# Peri-operative pharmacokinetics of cefazolin prophylaxis during valve replacement surgery

**DOI:** 10.1371/journal.pone.0291425

**Published:** 2023-09-20

**Authors:** Ahmad Alli, Fathima Paruk, Claire Roger, Jeffrey Lipman, Daren Calleemalay, Steven C. Wallis, Juan Scribante, Guy A. Richards, Jason A. Roberts

**Affiliations:** 1 Department of Anesthesiology and Pain Medicine, St Michael’s Hospital, University of Toronto, Toronto, Canada; 2 Faculty of Health Sciences, Department of Critical Care, University of Pretoria, Pretoria, South Africa; 3 Department of Anesthesiology, Critical Care Pain, and Emergency Medicine, Nimes University Hospital, Nimes, France; 4 University of Queensland Centre for Clinical Research, Faculty of Medicine, The University of Queensland, Brisbane, Queensland, Australia; 5 Department of Intensive Care Medicine, Royal Brisbane and Women’s Hospital, Brisbane, Queensland, Australia; 6 Faculty of Health Sciences, Department of Anesthesiology, University of Witwatersrand, Johannesburg, South Africa; 7 Surgeons for Little Lives and Department of Paediatric Surgery, School of Clinical Medicine, Faculty of Health Sciences, University of the Witwatersrand, Johannesburg, South Africa; 8 Faculty of Health Sciences, Division of Critical Care, University of Witwatersrand, Johannesburg, South Africa; 9 Centre for Translational Anti-infective Pharmacodynamics, School of Pharmacy, The University of Queensland, Brisbane, Queensland, Australia; 10 Pharmacy Department, Royal Brisbane and Women’s Hospital, Brisbane, Queensland, Australia; IRCCS Policlinico S.Donato, ITALY

## Abstract

**Objective:**

There is little prospective data to guide effective dosing for antibiotic prophylaxis during surgery requiring cardiopulmonary bypass (CPB). We aim to describe the effects of CPB on the population pharmacokinetics (PK) of total and unbound concentrations of cefazolin and to recommend optimised dosing regimens.

**Methods:**

Patients undergoing CPB for elective cardiac valve replacement were included using convenience sampling. Intravenous cefazolin (2g) was administered pre-incision and re-dosed at 4 hours. Serial blood and urine samples were collected and analysed using validated chromatography. Population PK modelling and Monte-Carlo simulations were performed using Pmetrics^®^ to determine the fractional target attainment (FTA) of achieving unbound concentrations exceeding pre-defined exposures against organisms known to cause surgical site infections for 100% of surgery (100% *f*T_>MIC_).

**Results:**

From the 16 included patients, 195 total and 64 unbound concentrations of cefazolin were obtained. A three-compartment linear population PK model best described the data. We observed that cefazolin 2g 4-hourly was insufficient to achieve the FTA of 100% *f*T_>MIC_ for *Staphylococcus aureus* and *Escherichia coli* at serum creatinine concentrations ≤ 50 μmol/L and for *Staphylococcus epidermidis* at any of our simulated doses and serum creatinine concentrations. A dose of cefazolin 3g 4-hourly demonstrated >93% FTA for *S*. *aureus* and *E*. *coli*.

**Conclusions:**

We found that cefazolin 2g 4-hourly was not able to maintain concentrations above the MIC for relevant pathogens in patients with low serum creatinine concentrations undergoing cardiac surgery with CPB. The simulations showed that optimised dosing is more likely with an increased dose and/or dosing frequency.

## Introduction

The risk of post-operative surgical site infection (SSI) is high following major cardiac surgery [1, 2]. Sternal wound infections, (including mediastinitis), endocarditis or prosthetic implant infections are of particular concern following cardiac surgery and are associated with poor patient outcomes and escalating costs of care [3]. The occurrence of mediastinitis in particular is a severe complication with mortality rates reported to be as high as 47% [4]. As such, effective antimicrobial prophylaxis is crucial.

Pathogens causing SSI, and their resistance patterns, are influenced by patient characteristics, pattern of antibiotic use and the effectiveness of infection control measures as well as geography. Although *Staphylococcus aureus*, *Staphylococcus epidermidis* and coagulase negative staphylococci are implicated in most SSIs, gram-negative pathogens such as *Pseudomonas* spp, *Acinetobacter* spp, *Enterobacteriaceae* and *Proteus mirabilis* are becoming increasingly responsible for complex SSIs [5]. Because gram positives, specifically *S*. *aureus* are the most common pathogens, cefazolin, a highly protein bound and hydrophilic first generation cephalosporin, is commonly recommended as the first line agent for antibiotic prophylaxis in cardiac surgery. An appropriate prophylactic regimen needs to cover the probable etiologic organisms and ensure that adequate concentrations are achieved at least from skin incision to closure. Defining an effective dosing regimen is particularly challenging when taking into account possible alterations in antibiotic pharmacokinetics (PK) related to surgery such as bleeding, hypotension and capillary leak and, perhaps even more so, the use of intraoperative cardiopulmonary bypass (CPB). CPB may lead to hemodilution, hypothermia, systemic inflammatory response, maldistribution of blood flow and the retention of the antibiotic within the extracorporeal circuit [6]. However, little prospective data are available to determine whether conventional cefazolin dosing regimens achieve optimal exposure during major cardiac surgery requiring CPB.

The aim of this study was to describe the population PK of cefazolin in a cohort of patients undergoing valve surgery with CPB. We then sought to use Monte Carlo simulations to propose optimized dosing regimens that have a high likelihood of achieving therapeutic exposures.

## Methods

This was a prospective, PK study with convenience sampling conducted in the cardiothoracic theatres of Charlotte Maxeke Johannesburg Academic Hospital, a quaternary academic training and referral center for cardiothoracic patients in South Africa. The University of the Witwatersrand Human Research Ethics Committee approved the study (clearance certificate No. M140662). Written informed consent was obtained from each participant.

### Patient population

Adult patients, aged between 18–60 years, undergoing CPB for elective cardiac valve replacement and receiving cefazolin prophylaxis were eligible for participation. Patients were excluded if they were pregnant, were Jehovah’s Witnesses, were receiving dialysis or had received cefazolin in the previous 72 hours.

### Conduct of study

Two grams of cefazolin was administered intravenously 30 minutes prior to skin incision. An additional 2 g was administered intraoperatively after 4 hours if surgery was ongoing, as recommended in the Society of Thoracic Surgeons (STS) guidelines [7, 8]. Blood samples were obtained from the central venous catheter (CVC) to determine total and unbound plasma cefazolin concentrations at baseline (pre-dose) and at 2, 5, 10, 30, 60, 90, 120, 150, 180, 210, 240, 270 and 300 minutes after cefazolin infusion. Blood samples were also obtained from the CPB circuit arterial port at 90, 120, 150, 180 min after cefazolin infusion. Urine samples were collected at the time of the urinary catheter insertion and then subsequently post completion of surgery. Demographic and clinical data, drug history, CPB time, surgical duration, preoperative renal function and albumin were recorded. There was no administration of pre-operative or post-operative antibiotics. CPB equipment, prime volume and parameters were selected by the attending perfusionist with the CPB flow rate (60-70ml/kg/min) and temperature (30–32 deg C) standardized.

#### Sample handling and storage

Blood samples were immediately placed on ice, centrifuged at 3000rpm for 10-minutes, and stored at -80°C in labelled microtubes. Plasma and urine samples were transported by a specialized commercial courier to the Burns Trauma and Critical Care Research Centre, University of Queensland Centre for Clinical Research, The University of Queensland, Australia, for bioanalysis.

#### Drug assay

Total and unbound cefazolin concentrations were measured by a validated method [9]: After thawing, the unbound fraction of cefazolin was isolated from the protein-bound fraction by ultracentrifugation of plasma. This was achieved at 37°C by centrifuging plasma with a Merck Millipore Centrifree device (30 KDa MWCO), with approximately 30% of the plasma centrifuged to avoid perturbing the equilibrium. We selected 3 plasma samples per patient for unbound concentration assay and these incorporated what we considered would be a high (2 mins post-dose), medium (30 mins post-dose) and low concentration (120-mins post dose). A UHPLC-MS/MS method was then used for concentration measurement; from 1 to 500 mg/L (total) and 0.1 to 500 mg/L (unbound), on a Shimadzu Nexera UHPLC connected to a Shimadzu 8030+ triple quadrupole mass spectrometer. Clinical samples were assayed alongside plasma calibrators and quality controls and met batch acceptance criteria (US FDA). Cefazolin concentrations in urine were measured by a validated UHPLC-MS/MS method from 100 to 10,000 mg/L [9].

The CPB extraction ratio (ER) was calculated using the following equation:

CPBER(%)=100X(preCPBcefazolinconcentration−postCPBcefazolinconcentration)/pre-CPBcefazolinconcentration.

The fraction of cefazolin cleared into urine (UrFE) over dosing interval (0–4 hours) was calculated as a function of the AUC in urine and plasma: UrFE = (AUC(0-4h) urine / AUC(0-4h) plasma) x 100.

#### Population pharmacokinetic modelling

To describe total and unbound cefazolin concentrations, two and three-compartment models were developed with the Nonparametric Adaptive Grid (NPAG) algorithm within the freely available Pmetrics software package for R (Los Angeles, CA) [10, 11].

Elimination of the total drug from the central compartment, inter-compartmental distribution into the peripheral compartment (three compartment model) and similarly inter-compartmental distribution into the unbound drug compartment (two and three compartment model) were modelled as first-order processes. Discrimination between different models used comparison of the -2 log likelihood (-2LL), Akaike Information Criteria (AIC) and Bayesian Information Criteria (BIC). A p-value of <0.05 was considered statistically significant.

#### Population pharmacokinetics covariate screening

Age, gender, body weight, body mass index (BMI), acute physiology and chronic health evaluation (APACHE) II score [12], serum creatinine concentration (SeCr), measured creatinine clearance, Cockcroft-Gault estimated creatinine clearance, serum albumin concentration, urine output, blood transfusion and pump prime fluid volume were evaluated as covariates. Selection of covariates for inclusion into the model was performed using a stepwise approach. Potential covariates were separately entered into the model and statistically tested by use of the -2LL, AIC and BIC values. If inclusion of the covariate resulted in a statistically significant improvement in the -2LL values (p<0.05) and/or improved the goodness-of-fit plots, then the covariate was retained in the final model.

#### Model diagnostics

Goodness of fit was assessed by linear regression, with an observed-predicted plot, coefficients of determination, and -2LL, AIC and BIC values. Predictive performance evaluation was based on mean prediction error (bias) and the mean bias-adjusted squared prediction error (imprecision) of the population and individual prediction models. The internal validity of the population PK model was assessed by the bootstrap resampling method (n = 1000) and normalized prediction distribution errors (NPDEs) [13]. Using visual predictive check (VPC) method, parameters obtained from the bootstrap method were plotted with the observed concentrations. NPDE plots were checked for normal distribution characteristics and trends in the data errors.

#### Probability of target attainment

Monte-Carlo simulations (n = 1000) were employed using Pmetrics to determine the probability of target attainment (PTA) of achieving 100% *f*T_>MIC_ for varying MICs (0.064 to 64 mg/L) during the first 4 hours of surgery for a standard patient with a serum creatinine concentration of 50 μmol/L and a serum albumin concentration of 35 g/L. A fixed 26% unbound fraction value of cefazolin was used for the simulations based on the measured unbound concentrations from the samples taken 120 minutes post-initial dosing. This was considered as the ‘worst-case scenario’ for the unbound fraction of cefazolin in this population. Intravenous doses of 1 g 4-hourly, 2 g 4-hourly, 3 g 4-hourly, 1 g 2-hourly, 2g 2-hourly, 2 g following by a subsequent dose of 1 g 2 hours later and 3 g followed by a dose of 1.5 g 2 hours later, were simulated. Three different levels of renal function were tested that reflected the broad distribution of values observed in this patient population (SeCr of 50, 75 and 100 μmol/L). Three different serum albumin concentrations (30, 35 and 40 g/L) were also simulated for a patient with a SeCr of 75 μmol/L.

#### Fractional target attainment calculation

MIC data of *S*. *aureus*, *S*. *epidermidis and Escherichia coli* from the EUCAST database were used to determine fractional target attainment (FTA) [14]. The fractional target attainment identifies the likely success of treatment by comparing the pharmacodynamic exposure (PTA) relative to the MIC distribution. The fractional target attainment was calculated using 100% *f*T_>MIC_. The PTA for achieving 100% *f*T_>MIC_ was calculated using Monte Carlo simulations (n = 1000) for various doses of 1 g 4-hourly, 2 g 4-hourly, 3 g 4-hourly, 1 g 2-hourly and 2 g 2-hourly during the first 4 hours of surgery for patients with SeCr of 50, 75, 100 μmol/L at MIC values from 0.064 mg/L to 64 mg/L. A specific dosing regimen was considered successful *a priori* if the FTA was ≥ 90%.

### Statistical analysis

Continuous data are presented as the mean (SD) or median [IQR]. Categorical data are presented as counts (%). Correlation was assessed by means of a scatter graph and Pearson correlation coefficient (r).

## Results

### Demographic data

Sixteen patients were included in the study with an equal number of males and females. The patients’ demographics are shown in Table 1.

**Table 1:** Patient demographics.

Demographics	Mean (SD)	Minimum	Maximum
Age (years)	44.0 (12.0)	18	59
Height (cm)	163.88 (9.98)	144	180
Weight (kg)	75.1 (15.6)	51	97
BMI (kg/m^2^)	28.22 (6.72)	18.1	40.2
Albumin (g/L)	41.6 (4.57)	33	47
Creatinine (μmol/L)	76.7 (18.6)	42	102
Estimated creatinine clearance (ml/min) ^a^	81.5 (25.8)	42.5	124.63
CBP duration (min)	165 (52.6)	105	278
Pre-CPB infusion (ml)			
• Crystalloid^b^	5319 (2149.2)	2100	11500
• Allogeneic blood	500 (311.8)	250	1250
Post-CPB infusion (ml)			
• Crystalloid	418 (235.9)	100	1000
• Allogeneic blood	393 (133.6)	250	500
Urine output (ml)	1081.3(963.5)	200	3600

BMI–body mass index; CBP–cardiopulmonary bypass

^a^ Estimated according to Cockcroft-Gault equation

^b^ Includes priming of CPB circuit

### Population pharmacokinetic model

**Fig 1:**
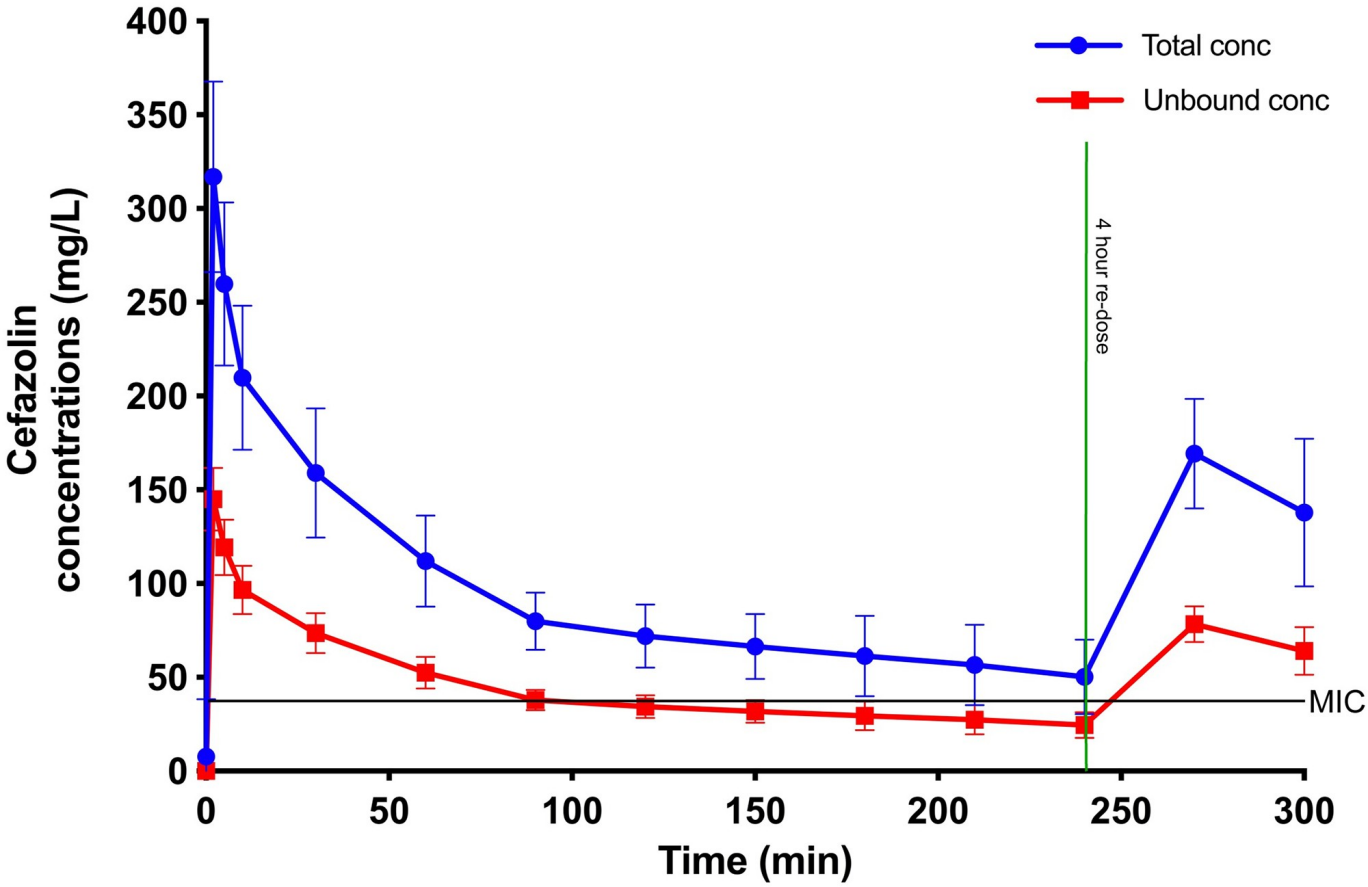
The observed mean total (black) and unbound (grey) cefazolin concentration-time profiles in cardiopulmonary bypass (CPB) surgical patients (n = 16). This figure demonstrates the fall of cefazolin concentration below the optimal minimum inhibitory concentration (MIC) after 200 minutes. Error bars represent standard deviation. Conc: Concentration.

The mean observed concentration-time profile of the unbound and total cefazolin concentrations is shown in Fig 1.

**Fig 2:**
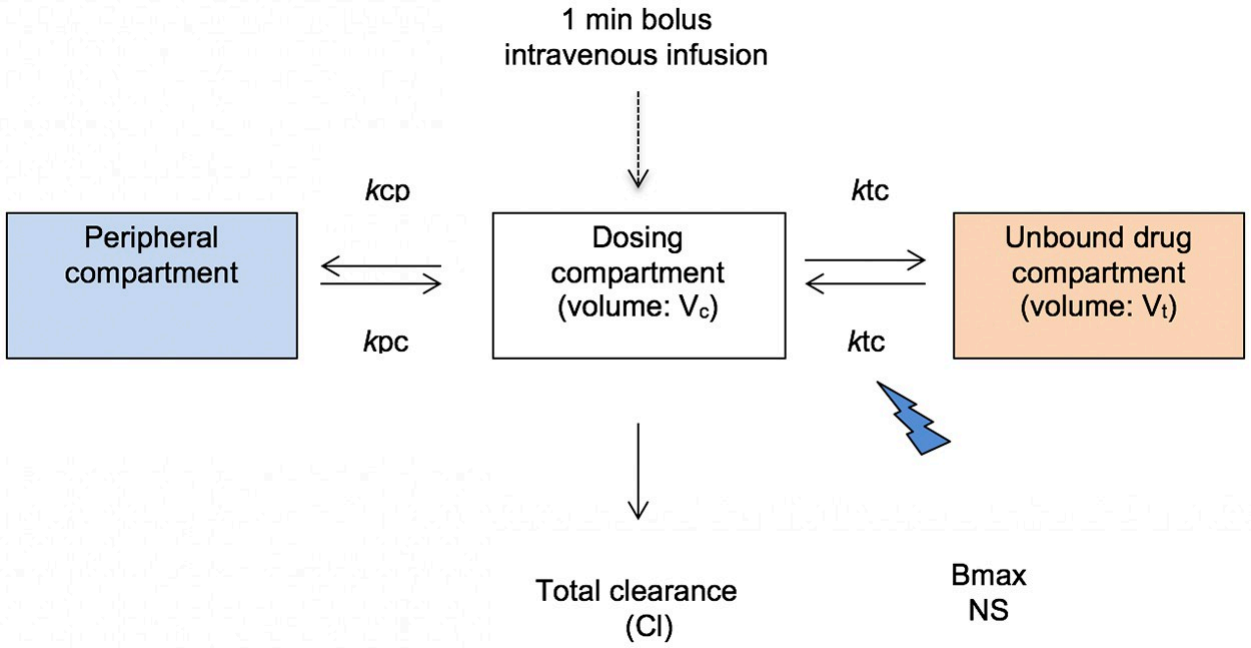
Structural pharmacokinetic model for cefazolin in CPB surgical patients. *kcp*–rate constant for drug distribution from the central to peripheral compartment; *kpc*–rate constant for drug distribution from the peripheral to central compartment; *kct*–rate constant for drug distribution from the central to unbound drug compartment; *ktc*–rate constant for drug distribution from the unbound drug to central compartment; Bmax–maximal binding capacity; NS–non saturable protein binding.

Although a total of 288 samples were potentially collectable for processing, due to variable CPB and surgery times,195 samples were available for total concentration analysis. Following the methodology described above, 64 unbound concentrations were analyzed. A three-compartment linear model best described the time-course of cefazolin (Fig 2). This model included zero order input of drug into the central compartment.

The only covariate that improved the fit of the model was, for cefazolin clearance, a serum creatinine concentration normalized to the population mean value of 90 μmol/L and, for protein binding a serum albumin concentration normalized to the population mean value of 40 g/L (p<0.05). After including these parameters the -2LL values decreased (-17 Δ-2LL, p = 0.02) and the goodness-of-fit improved. As such, serum creatinine concentration and albumin were retained in the final model.

A multiplicative error equation was supported for use in this model with the equation:

$$\text{Error}={\left(\text{SD}*\gamma \right)}^{2},$$

where SD represents the standard deviation of observations and γ represents process noise.

In addition, assay error for plasma data was included as:

Error=C0+C1*obs,

where obs represents observations; and coefficients C0 (limit of quantification) and C1 (% coefficient of variation of assay) were included as 1.0 mg/L and 0.1 respectively. For unbound data, C0 and C1 were 0.1 and 0.1 respectively.

The final clearance model was described as follows: Cefazolin CL = TVCL*(90/SeCr) where TVCL is the typical value of cefazolin clearance and SeCr is the serum creatinine concentration in μmol/L.

The protein binding was described as follows: PB = (*k*tc*Bmax+NS)*(ALB/40) where PB is the rate constant for protein binding from unbound to total drug; *k*tc is the rate constant for drug distribution of the unbound drug to central compartment; Bmax is the maximal binding capacity; NS is non-saturable protein binding; ALB is the serum albumin concentration in g/L.

The measures of central tendency (mean and median) and dispersion (SD) of the population PK parameter estimates from the final covariate model are shown in Table 2. Mean clearance of cefazolin was 3.23 L/h (± 1.16), central volume(Vc) was 3.38L (±0.73), and the unbound volume of the unbound drug compartment was 6.08L (±2.03). Non-saturable protein binding was 15.14(±3.75). Peripheral volume of distribution was calculated manually using the equation: Vp = Vc*kcp/kpc where Vp is peripheral volume; kcp is the rate constant for drug distribution from the central to peripheral compartment; and kpc is the rate constant for drug distribution from the peripheral to the central compartment. The mean total volume of distribution for this study was 8.9L. The diagnostic plots to confirm the goodness of fit of the model were considered acceptable (S1 and S2 Figs). The final covariate model was then used for dosing simulations.

**Table 2:** Parameter estimates for cefazolin from the final covariate 3-compartment population pharmacokinetic model.

Parameters	Mean	Standard deviation	Coefficient of variation	Variance	Median
Cl (L/h)	3.23	1.16	36.06	1.36	3.00
Vc (L)	3.38	0.73	21.59	0.53	3.41
Vt (L)	6.08	2.03	33.42	4.13	6.28
*k*cp (h^-1^)	5.92	3.88	65.58	15.05	4.59
*k*pc (h^-1^)	2.24	1.16	51.81	1.35	2.35
*k*ct (h^-1^)	144.27	14.67	10.17	215.30	143.84
*k*tc (h^-1^)	195.91	25.34	12.94	642.33	188.90
Bmax (g/L)	1.01	0.46	45.23	0.21	0.87
NS	15.14	3.75	24.79	14.08	15.20

Cl–Clearance; Vc–central volume; Vt–volume of unbound drug compartment; *kcp*–rate constant for drug distribution from the central to peripheral compartment; *kpc*–rate constant for drug distribution from the peripheral to central compartment; *kct*–rate constant for drug distribution from the central to unbound drug compartment; *ktc*–rate constant for drug distribution from the unbound drug to central compartment; Bmax–maximal binding capacity; NS–non saturable protein binding.

Based on urine cefazolin concentrations, the UrFe for the dosing interval was 39.6% (± 0.2%). The mean CPB ER of cefazolin was 3.2% (± 2.1%).

### Dosing simulations

**Fig 3:**
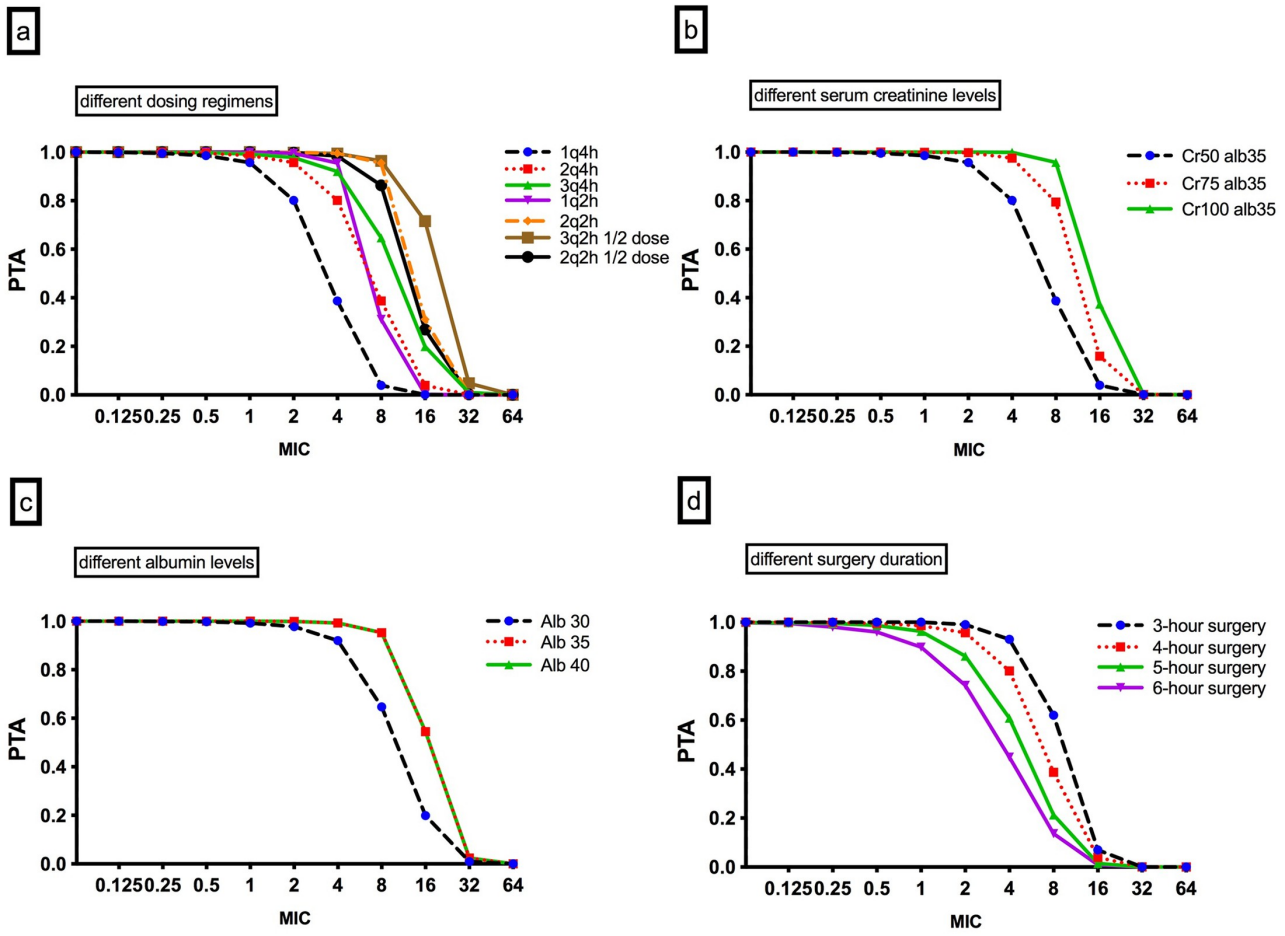
Monte Carlo simulations and PTA (*f*T/MIC = 100%) in plasma against various MICs for various IV cefazolin doses for: (a) a standard patient with a serum creatinine level of 50 μmol/L and a serum albumin level of 35 g/L. (b) a 2 g intravenous 4-hourly cefazolin dose for different levels of serum Creatinine. (c) a 3 g intravenous 4-hourly cefazolin dose for different serum albumin Levels. (d) a 2 g intravenous 4-hourly cefazolin dose for different durations of surgery This figure shows that the PTA is more optimal with higher or more frequent cefazolin dosing, greater creatinine values, higher albumin and shorter surgical duration. PTA: probability of target attainment, MIC: Minimum inhibitory concentration, 1g4h: 1 gram 4 hourly, 2g4h: 2 gram 4 hourly, 3g4h: 3 gram 4 hourly, 1g2h: 1 gram 2 hourly, 2g2h: 2 gram 2 hourly, 3g2h ½ dose: 1.5 gram 2 hourly, 2g2h ½ dose: 1g 2 hourly, Cr50: At a creatinine level of 50μmol/L, Cr75: At a creatinine level of 75μmol/L, Cr100: At a creatinine level of 100 μmol/L, Alb35: At an albumin concentration of 35g/L, Alb30: At an albumin concentration of 30g/L, Alb40: At an albumin concentration of 40g/L.

The PTAs for various cefazolin doses for a patient with a serum creatinine concentration of 50 μmol/L and a serum albumin concentration of 35 g/L are presented in Fig 3a. They illustrate that the standard regimen (2 g 4 hourly) was inadequate for a MIC of 4 mg/L during the first 4 hours of surgery. The simulations also showed that increasing dose and/or increasing dose frequency resulted in an increased PTA. Fig 3b and 3c describe the effect of different serum creatinine (b) and serum albumin (c) concentrations on PTA. Normal or elevated serum creatinine concentrations were associated with a greater likelihood whilst lower than normal albumin concentrations were associated with a reduced PTA at a MIC of 4 mg/L.

### Fractional target attainment

The FTA for the simulated PTAs for a range of cefazolin doses and serum creatinine concentrations against MIC distributions in the susceptible range for *S*.*aureus*, *S*.*epidermidis* and *E*. *coli* are shown in Table 3. The dosage regimen of 2 g cefazolin re-dosed 4 hourly was suboptimal for all pathogens at creatinine concentrations ≤ 50 μmol/L for surgery of 4 hour duration and for *S*.*epidermidis* regardless the dose. Although some regimens that were considered sub-optimal (under 90%) were within 1% of the acceptable threshold, the use of 1000 simulations across a large isolate bank (*S*. *aureus* 19,252 isolates; *S*. *epidermidis* 1498; *E*. *coli* 285 isolates) supports use of one decimal point.

**Table 3:** Fractional target attainment of 100% *f*T_>MIC_ for the various cefazolin doses for patients with serum creatinine (SeCr) of 50, 75 and 100 μmol/L, albumin level of 35 g/L and a duration of surgery of 4 hours for a *S*. *aureus*, *S*. *epidermidis*, *E*. *coli* MICs distribution.

SeCr (μmol/L)	Dosing regimen	*S*. *aureus*	*S*. *epidermidis*	*E*. *coli*
50	1g4h	86.8%	60.5%	78.6%
	2g4h	89.6%	72.7%	89.8%
	3g4h	90.5%	77.8%	93.2%
	1g2h	90.4%	75.0%	92.6%
	2g2h	91.3%	82.2%	96.4%
	2g/1g 2h	91.2%	81.1%	95.8%
	3g/1.5g2h	92.0%	85.1%	97.2%
75	1g4h	89.9%	71.2%	89.8%
	2g4h	91.0%	79.8%	95.2%
	3g4h	91.7%	83.8%	96.8%
	1g2h	90.9%	79.4%	95.2%
	2g2h	91.8%	85.4%	97.5%
100	1g4h	90.5%	75.6%	93.0%
	2g4h	91.4%	82.6%	96.6%
	3g4h	92.3%	86.5%	97.7%
	1g2h	91.1%	80.6%	95.9%
	2g2h	92.0%	86.5%	97.8%

Doses not achieving the a priori probability of target attainment (PTA) of 100% *f*T_>MIC_ against at least 90% of isolates are shaded grey.

## Discussion

In this population PK study in patients undergoing CPB surgery, we found that the current regimen of cefazolin (2 g 4-hourly) was insufficient to achieve the PK/PD target of 100% *f*T_>MIC_ for relevant pathogens during the first 4 hours of open cardiac surgery in patients with normal renal function and serum albumin concentrations. In line with the pharmacodynamics of beta-lactams, our dosing simulations showed that increasing the dose and/or increasing dosing frequency resulted in improvements in the PTA and FTA.

The suboptimal achievement of PK/PD targets with the 2g 4-hourly dosing observed in this study was not unexpected. CPB generally occurs in the setting of a multitude of other insults including the trauma of surgery, exposure to foreign materials, ischemia-reperfusion injury and altered shear stresses during blood flow and hypothermia [15]. A significant consequence of the inflammatory response is increased capillary permeability and falling serum albumin concentrations. These factors may result in higher unbound concentrations which distribute more widely, significantly increasing the Vd of drugs like cefazolin. An increased unbound concentration, as was observed in this study, may be initially advantageous however, it may be more easily redistributed and renally cleared, leading to low antibiotic concentrations later in the dosing interval [16]. Interestingly in our study, estimates of cefazolin clearance were mildly lower than that from other studies, in spite of quite a high unbound fraction of drug [17, 18].

There is limited data pertaining to cefazolin PK during CPB [19, 20] and for other commonly prescribed prophylactic antibiotics such as cefuroxime [21], vancomycin [22] and teicoplanin [23]. Nonetheless, these data suggest that antibiotic doses used for peri-operative prophylaxis of infection may require re-evaluation due to a shortened half-life, due to the factors described above. Lanckhor and colleagues have recently reported a significant effect of CPB on cefazolin Vd and recommended that it should be re-dosed after CPB initiation [24]. Caffarelli and coworkers [20] demonstrated that a prolonged duration (> 120 min) of CPB predisposed about 50% of patients to manifest sub-therapeutic concentrations following a dosing regimen of 1 g cefazolin post induction of anaesthesia followed by a second 1 g dose before wound closure. Fellinger and colleagues [19], demonstrated that despite additional intraoperative cefazolin (i.e. 1 g on induction of anaesthesia plus 1 g after CPB initiation) serum concentrations did not achieve targets for *Enterobacter spp*., *Serratia spp*., *E*. *coli*, and *Proteus mirabilis*. More recently Zelenitsky et al. demonstrated in a retrospective evaluation of patients undergoing cardiac surgery under CPB, that lower cefazolin concentration at sternal closure and longer duration of surgery were associated with an increased risk of SSI [25]. In addition, the authors suggest that the threshold concentration of cefazolin required to be effective against SSIs, is greater than 104 mg/L (free concentration: 29 mg/L), higher than the accepted EUCAST values.

In order to mitigate anticipated reductions in antibiotic concentrations, a supplemental dose of prophylactic antibiotics is usually given within the CPB circuit or upon completion of CPB [26, 27]. This may, because of individual dosing requirements, actually exceed the target exposure and there is little evidence to suggest that this is likely to be harmful [28, 29].

Reduced serum creatinine concentrations and hypo-albuminemia were associated with a reduced likelihood of PTA at a MIC of 4 mg/L for the 2g dose. These scenarios are frequently encountered in this population and thus need to be considered when determining the appropriate dose and frequency of cefazolin. Given that cefazolin is hydrophilic and predominantly renally excreted, patients with renal dysfunction are likely to exhibit reduced antibiotic clearance. Conversely patients with higher glomerular filtration rates (GFRs) and lower serum creatinine concentrations may manifest a higher clearance of cefazolin, the so called augmented renal clearance. In our study SeCr ranged from 42 to 102 umol/L. Significantly, we found that the dosing regimen we used was inadequate in patients with SeCr under 50 umol/L.

Whilst obesity did not significantly influence cefazolin clearance in this cohort, it has been cited as an important consideration elsewhere [30].

The mean CPB ER of cefazolin of 3.2% is low. Studies have demonstrated circuit sequestration of both antibiotic during CPB procedures [31, 32]. The degree of sequestration for most antibiotics is unknown and may also depend on the nature of the circuit (e.g. material, surface area).

### Implications of the study findings

It is recognised that CPB can increase cefazolin Vd as a result of various factors associated with the use of hemodilution, altered protein binding, tissue distribution, and sequestration in the CPB circuit. Given that cefazolin is a hydrophilic drug, the magnitude of the effect of CPB on cefazolin PK may be significant. Some uncertainty remains regarding the optimal dosing schedule patients undergoing surgery requiring CPB. The findings of this study suggest that the recommended dosing regimen of 2 g 4-hourly is adequate for the relevant pathogens in patients with reduced renal function. However, in patients with low serum creatinine concentrations and normal albumin concentrations, higher than recommended dosing regimens, i.e., increased dose (3g 4-hourly) or dose frequency (2g 2-hourly), should be used.

Alternative dosing strategies such as the use of a bolus dose followed by a continuous infusion could also be effective [33, 34]. Further studies are required to assess these strategies in CPB surgery.

### Weaknesses in the study

The sample size may not have been completely sufficient to describe full population PK variability. A larger sample size may have enabled the identification of more covariates associated with altered cefazolin PK in these patients. However, sample sizes of this size are considered acceptable in similar PK/PD studies consisting of intensively sampled analyses [35]. Although we believe that a reduction of SSI would be seen by targeting MICs as set out by EUCAST, this study was a PK analysis and no postoperative clinical outcomes such as SSI were recorded. Serum creatinine was used instead of creatinine clearance as the serum creatinine value is often used in clinical contexts to determine prophylactic cefazolin dosing. Serum creatinine values do not consider patient characteristics such as age and weight, and therefore isolated serum creatinine values may be misleading. However, patient age or weight were not significant covariates, and we believe that the current model is statistically robust and clinically applicable. Lastly, the findings from this study are contextual to our population, surgical and CPB techniques, and may not be able to be generalized. Choice of the cardiopulmonary bypass circuit and components were left to attending perfusionists, and differing component materials and surface areas could have resulted in variable CPB circuit sequestration. In addition, the EUCAST distribution in the analysis includes both susceptible and non-susceptible pathogens. Following PTA results with the use of local MIC data is advisable for a more localized representation of microbial susceptibility and antibiotic dosing requirements.

## Conclusion

The dosing regimen of intravenous administration of 2g cefazolin 4-hourly, was inadequate for achieving target drug exposures for a standard patient, undergoing valve surgery receiving CPB, with a serum creatinine (SeCr) level of 50 μmol/L and an albumin concentration of 35 g/L. The efficacy of regimens with either a higher dosage or increased frequency or the use of continuous infusions needs to be explored in this population. In order to ensure successful prophylaxis, existing dosing regimens need to be re-evaluated and individualised, particularly taking patient factors such as renal function into account.

## Supporting information

S1 Fig Diagnostic plots for the final covariate model. Observed versus population predicted concentrations (left-hand panels) and individual predicted concentrations (right-hand panels) for total (a) and unbound (b) concentrations. Data are presented in mg/L.
(TIF)

S2 Fig Visual predictive check of total plasma data. Blue circles represent observed data.
(TIF)
