# Supplementary figures and images for: Peri-operative pharmacokinetics of cefazolin prophylaxis during valve replacement surgery
Source: PLoS One. 2023 Sep 20;18(9):e0291425. doi: 10.1371/journal.pone.0291425 (PMC10511078; doi:10.1371/journal.pone.0291425)

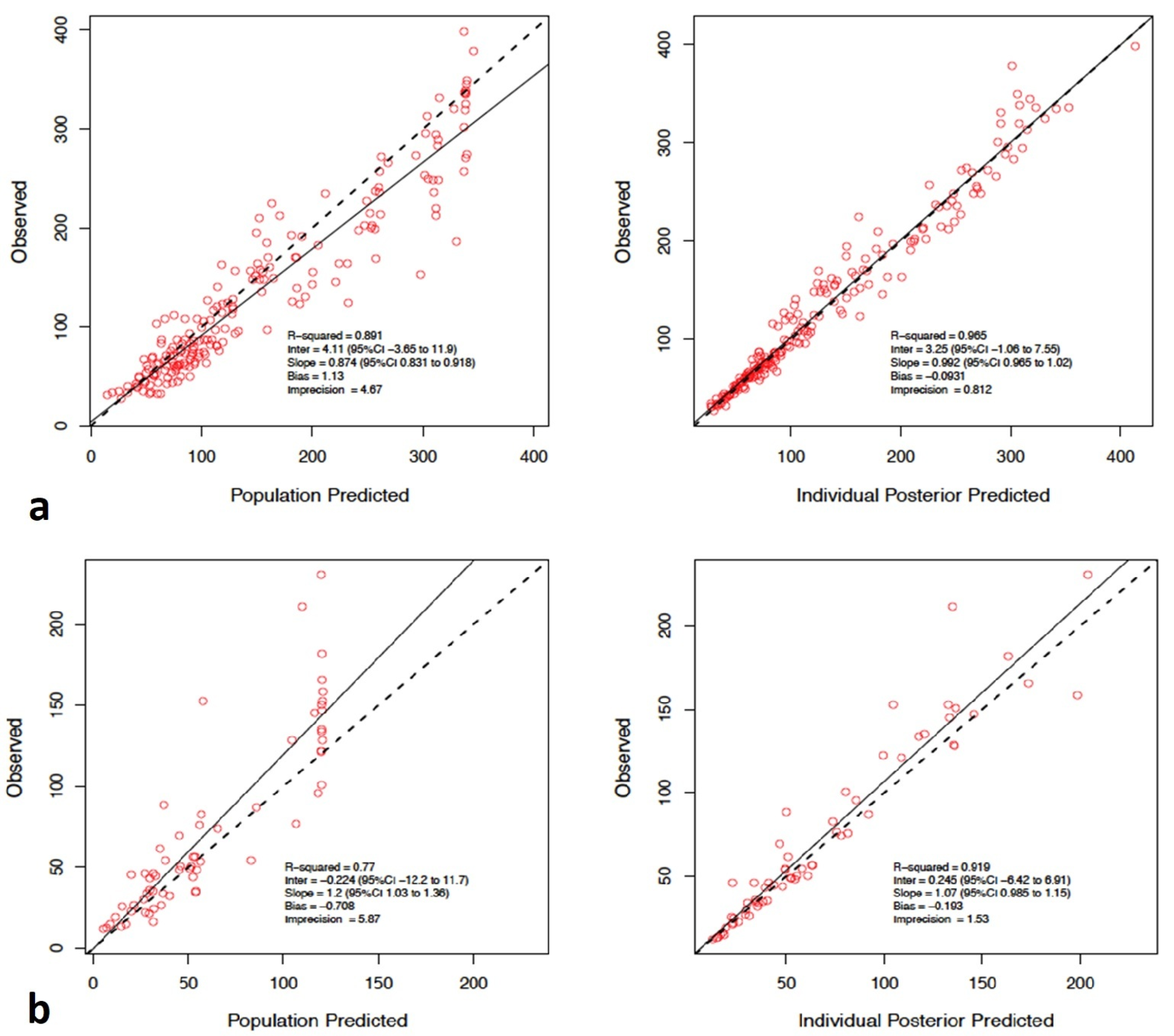

Supplement: S1 Fig — Observed versus population predicted concentrations (left-hand panels) and individual predicted concentrations (right-hand panels) for total (a) and unbound (b) concentrations. Data are presented in mg/L. (TIF) [file pone.0291425.s001.tif]

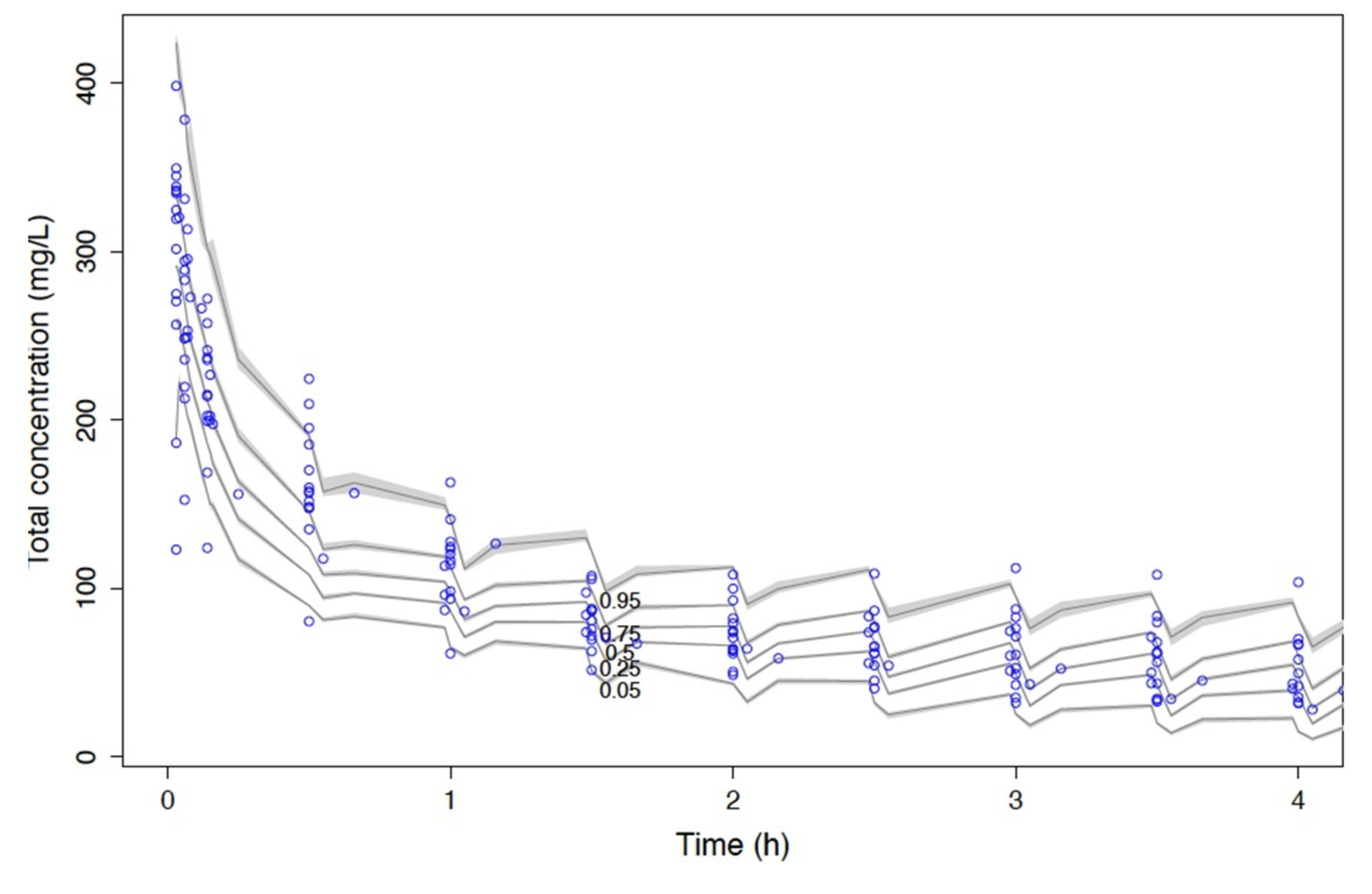

Supplement: S2 Fig — Blue circles represent observed data. (TIF) [file pone.0291425.s002.tif]
